# Precise and Prompt Analyte Detection via Ordered Orientation of Receptor in WSe_2_-Based Field Effect Transistor

**DOI:** 10.3390/nano12081305

**Published:** 2022-04-11

**Authors:** Muhammad Shahzad Zafar, Ghulam Dastgeer, Abul Kalam, Abdullah G. Al-Sehemi, Muhammad Imran, Yong Ho Kim, Heeyeop Chae

**Affiliations:** 1 School of Chemical Engineering, Sungkyunkwan University, Suwon 16419, Korea; mshahzad@skku.edu (M.S.Z.); hchae@skku.edu (H.C.); 2 SKKU Advanced Institute of Nanotechnology (SAINT), Sungkyunkwan University, Suwon 16419, Korea; yhkim94@skku.edu; 3 Department of Physics and Astronomy, Graphene Research Institute-Texas Photonics Center International Research Center (GRI–TPC IRC), Sejong University, Seoul 05006, Korea; 4 Department of Chemistry, Faculty of Science, King Khalid University, P.O. Box 9004, Abha 61413, Saudi Arabia; abul_k33@yahoo.com (A.K.); agmsq@kku.edu.sa (A.G.A.-S.); imranchemist@gmail.com (M.I.); 5 Research Center for Advanced Materials Science (RCAMS), King Khalid University, P.O. Box 9004, Abha 61514, Saudi Arabia

**Keywords:** tungsten di-selenide, gate-tunable, biosensor, orientation control, protein detection

## Abstract

Field-effect transistors (FET) composed of transition metal dichalcogenide (TMDC) materials have gained huge importance as biosensors due to their added advantage of high sensitivity and moderate bandgap. However, the true potential of these biosensors highly depends upon the quality of TMDC material, as well as the orientation of receptors on their surfaces. The uncontrolled orientation of receptors and screening issues due to crossing the Debye screening length while functionalizing TMDC materials is a big challenge in this field. To address these issues, we introduce a combination of high-quality monolayer WSe_2_ with our designed Pyrene-based receptor moiety for its ordered orientation onto the WSe_2_ FET biosensor. A monolayer WSe_2_ sheet is utilized to fabricate an ideal FET for biosensing applications, which is characterized via Raman spectroscopy, atomic force microscopy, and electrical prob station. Our construct can sensitively detect our target protein (streptavidin) with 1 pM limit of detection within a short span of 2 min, through a one-step functionalizing process. In addition to having this ultra-fast response and high sensitivity, our biosensor can be a reliable platform for point-of-care-based diagnosis.

## 1. Introduction

For a better health monitoring and curing system, the precise and prompt detection of target analytes has gained importance. As in the COVID-19 situation, the rapid screening of COVID-19 patients by sensitively detecting the spike protein can prevent the spread of the virus [1,2]. Hence, there is high demand for a point-of-care (POC)-based health monitoring system which can offer an ideal platform with low-cost, reliable, and easy-to-use diagnostics of key biomarkers needed for the early screening of pandemics and other health concerns. Most established methods for protein-based molecular diagnostic tests, such as immunoassays, are time-consuming and costly to use, since they require many reagents and skilled personnel. Due to this, their implementation as a POC-based system has been limited and has become partially ineffective for service in a pandemic situation [3]. Hence, substrate-based detection systems are needed, as they provide the lab-on-a-chip platform. However, the currently established substrate-based protein detection systems are facing challenges regarding uncontrolled orientation, stabilization of target protein with good surface density, and selectivity [4,5,6]. In short, a substrate based POC device with accurate, real-time, and rapid response against target protein is urgently needed. Researchers have made tremendous efforts to develop highly sensitive substrates by using various 2D materials as channels, such as transition metal dichalcogenide-based field-effect transistors (TMDC-FET). Several practical devices such as field-effect transistors (FETs), solar cells, logic circuits, memories, diodes, photo detectors, etc. have been prepared based on single- or van der Waals stack of two same or different TMDs for effective operations and controlled functionalities [7,8,9]. These materials are preferred on simple graphene or graphene oxide due to their high sensitivity, inherent bandgap, and compatibility with planner surfaces. Among the atomically layered TMDCs, molybdenum disulfide (MoS_2_) and tungsten diselenide (WSe_2_) have been widely studied as attractive materials for biosensing applications [10,11,12,13]. Compared to pure graphene-based FETs, TMDS materials have a modest bandgap, which reduces leakage current significantly during device measurements, resulting in better sensitivity [14,15]. However, the WSe_2-_based FET biosensor exhibited better linear regime sensitivity than MoS_2_-based FETs for the detection of tumor necrosis factor-alpha detection [16]. This better performance is attributed to the ambipolar transfer characteristics of WSe_2_ FET and relatively better carrier mobility [17,18]. Most recently, the WSe_2_-based FET device has been utilized for early stage detection of prostate cancer protein [19].

However, the practical application of WSe_2_ FETs has been limited due to various bio-physical environment issues such as the uncontrolled orientation of receptors. Due to these issues, most of the sensor surface remains blocked and unavailable for detecting biomolecules, resulting in loss of sensitivity. Moreover, it may lead to surface aggregations and non-specific bindings of biomolecules to the substrate, creating wrong signal readouts [20]. In addition to the above-mentioned issues, the design of receptors themselves can sometimes cause loss of sensitivity due to various screening effects, as the sensor can only efficiently detect the binding events occurring within the Debye screening length [21,22,23,24]. Researchers try to avoid some of the challenges through smart device operation, i.e., by applying high gate voltages to bias the FET for high sensitivity [16,25]. However, these electrical stresses may cause high leakage currents, resulting in the damaging of devices and making them unfeasible for long time-dependent measurements [22,26]. Furthermore, researchers have put forth a lot of work to control the orientation of receptors on the sensor surface for sensitive target analyst detection. As an example, various covalent bindings can be used to regulate the orientation of receptors on the substrate. They include amine and carboxylic group EDC/NHS couplings [27] and thiol bindings to Au-coated electrochemiluminescence probes [28]. These functional groups can be found on the outside of proteins or antibodies. In this regard, a WSe_2_-based FET biosensor based on EDC/HNS coupling of 11-mercaptoundecanoic acid has been reported for the detection of spike protein for COVID-19 detection [29]. In addition to the aforementioned methods, researchers have attempted to replace the surface assembly monolayer (SAM) by oxidizing the carbohydrates found in antibodies’ Fc region [30]. However, site-controlled covalent immobilization is difficult to produce, and the above-mentioned methods’ sensitivity has been limited due to potential conformational difficulties and surface aggregation. A nano-gap sensor device is another intriguing concept for controlling the orientation and avoiding surface aggregation. The sensor detects changes in ionic currents through a nanopore in a graphene membrane using hydrophobic interactions between graphene and the target material [31]. The method, however, can only be used to detect small molecules such as DNA fragments.

Researchers have attempted to control orientation using Pyrene-linked aptamer [32] and polymers [33] in the use of an electrochemical biosensor. The use of Pyrene-based receptors for the directed functionalization of graphene substrate in FETs has previously been reported [34,35]. However, there are still issues due to the temperature sensitivity of polymers and the possibility of steric hindrance induced by various non-specific bindings to the sensor surface. Surface aggregations can also occur as a result of big proteins or antibodies binding to the Pyrene moiety, which is relatively small (0.3 nm). Additionally, the use of protein G-DNA conjugate for DNA-directed antibody immobilization has been described [36]. However, due to the possibility of surface aggregation and diverse screening effects, the system’s performance suffered.

Furthermore, self-association of naturally occurring or de novo designed proteins or peptides has been used for the directional functionalization of substrates to improve their diagnostic potential [37]. The use of S-layer proteins, in particular, is noteworthy [38]. The size of these proteins and peptides, however, can reach up to 20 nm, causing numerous screening challenges during device measurements. As a result, due to the complicated bio-physical system, functionalizing the surface with good surface density and selectivity remains a major difficulty.

Here, we present a strategy to conserve the sensitivity of WSe_2_-based FET by improving the quality of WSe_2_ material and designing a receptor to ensure its ordered orientation onto the WSe_2_ channel upon functionalization. Our designed receptor makes its way to attach to the WSe_2_ surface by π-π stacking and detects the target analyte by ruling out the screening effects. For a practical demonstration of our developed WSe_2_ FET biosensor, we utilized a well-known biotin-SA system by conjugating biotin on our Pyrene-based receptor via solid-phase peptide synthesis (SPPS). Our system can selectively encounter the picomolar level of streptavidin (SA) within less than 2 min. Device selectivity was proved by measuring the current response of WSe_2_ FET towards serum protein (BSA) of similar (66.46 kDa) molecular weight with our target protein (66 kDa). According to the findings, our suggested technique can accurately and quickly detect any protein of interest. The astonishing results indicate the possibility of producing devices for biosensing that are both smart and efficient with the ability to operate at a low power.

## 2. Materials and Methods

### 2.1. Device Fabrication

First, the SiO_2_ coated *p*-silicon substrates are cleaned with Acetone and methanol and dried with the Nitrogen gas. The SiO_2_/*p*-Si substrates are treated under deep-ultra-violet (DUV, Bachur & Associates, Santa Clara, Canada) light and Oxygen plasma for 5 min to remove the residue and make the substrates ultra clean. Then, the High-quality bulk crystals of WSe_2_ material (purchased from the HQ Graphene manufacturer company, Groningen, The Netherlands) are mechanically exfoliated using a piece of scotch tape. The thin layers of the WSe_2_ are cleaved and directly transferred over the SiO_2_/*p*-Si substrate. The monolayer sheets of WSe_2_ are traced by optical microscopic color contrast and confirmed via atomic force microscopy (AFM, I-Nexus Co., Ltd., Seoul, Korea) analysis for the device fabrication process. To fabricate a clean and residue-free FET, all the devices are annealed at 200 °C for two hours in an Argon gas atmosphere before patterning the metal electrodes. Secondly, the photolithography technique is utilized to pattern the big metal electrodes around the WSe_2_ sheet. The substrates loaded with the WSe_2_ monolayer sheets were coated with polymer EL-9 and photo resist, placed under a shadow mask, and exposed under the DUV light for big patterns. Then, using thermal deposition in an ultra-high vacuum, large metal electrodes (Ti/Au) were deposited in a metal deposition chamber.

**Figure 1:**
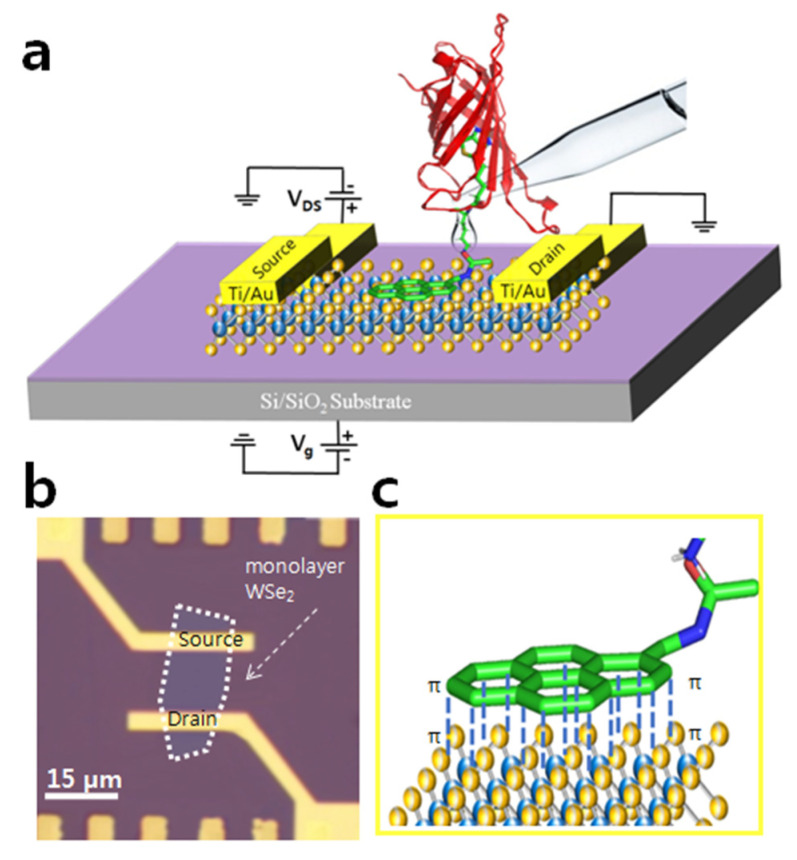
Schematic illustration of WSe_2_ based FET device for protein detection. The device utilizes protein–protein interaction and high sensitivity of WSe_2_. (**a**) The device construct having monolayer WSe_2_ on Si/SiO_2_ substrate. After directionally functionalizing the device with our designed Pyrene-based supporter molecule, the solution containing the target protein (SA) was drop casted onto the device for its detection. The level of current depicts various stages of device operation. The highlighted part represents the insights of surface chemistry where Pyrene moiety binds to the channel via π-π stacking (binding energy of ~2 kcal mole^−1^). (**b**) Optical image of the monolayer WSe_2_. (**c**) The schematic image is showing the π-π stacking of the Pyrene ring with the WSe_2_ sheet.

Finally, the scanning electron microscope (SEM, TESCAN, Ltd., Seoul, Korea,) assisted with the Quantum Alpha was used for the electron beam lithography to pattern the microelectrodes over the WSe_2_ sheets. After electron beam lithography, the samples were loaded in the metal deposition chamber again to depose the metal electrodes (Ti/Au) over the monolayer WSe_2_ sheet via thermal deposition. To enhance the device sensitivity and to avoid the Schottky contribution, the Ti/Au electrodes are deposited over the WSe_2_ [39,40,41,42]. The schematic diagram and optical image of the final device are illustrated in Figure 1a,b.

**Figure 2:**
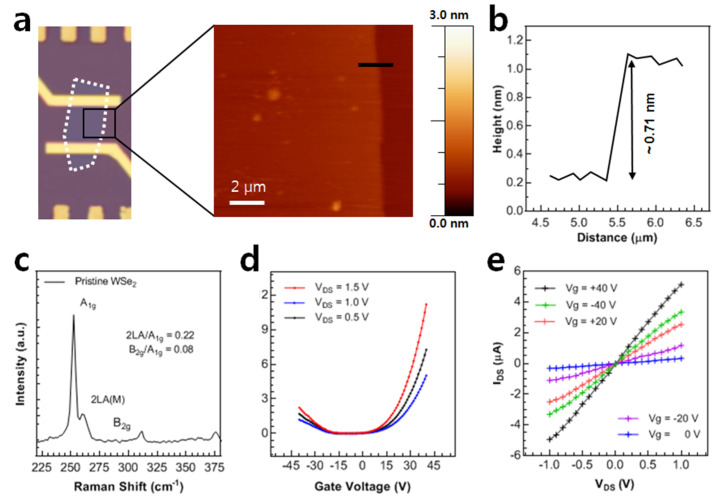
Material characterization and electrical properties of pristine WSe_2_ FET. (**a**) The surface analysis and sheet thickness of WSe_2_ semiconducting channel via AFM analysis. (**b**) The uniform thickness of ~0.71 nm confirms the monolayer of WSe_2_. (**c**) Raman spectra of the monolayer WSe_2_. The small peak ratios of resonance and defects confirm the monolayer and crystallinity of WSe_2_. (**d**) The transfer characteristics of the pristine WSe_2_ at various V_DS_. The threshold gate voltages of >10 and <–20 represent the dominant carrier density of electrons (*n*-type behavior). (**e**) The output characteristics of the pristine WSe_2_ at various gate voltages. The suppressed current at negative gate voltage depicts the *n*-type nature of the WSe_2_ sheet.

Surface analysis and the thickness of the monolayer WSe_2_ sheet were confirmed by atomic force microscopy (AFM) analysis. Figure 2a shows an AFM image and a height profile of the WSe_2_ sheet from the mentioned area of the black square box, confirming the monolayer WSe_2_ sheet with a thickness equivalent to 0.71 nm. Moreover, the Raman analysis presented in Figure 2b also confirms that the thickness of the WSe_2_ sheet corresponds to nearly one layer. The stepwise fabrication of the monolayer WSe_2_ FET device and its application to detect the protein molecules is illustrated in the Supporting Information in Figure S1.

### 2.2. Supporter Synthesis

The supporter molecule (biotin_ Lysine_ Pyrene construct) was synthesized by a manual solid-phase peptide synthesizer (SPPS), using Fmoc chemistry-based H-Rink amide ChemMatrix resin (0.54 mmol/g substitution value, PCAS BioMatrix Inc., Louisville, KY, USA). The 250 mg of resin (estimated based on a 0.14 mmol synthesis scale) was swelled in DMF for 30 min before starting synthesis. For de-protection, 20% piperidine in N, N-dimethylformamide (DMF), and the reaction mixture were heated at 75 °C ± 5 for 15 s and 90 °C ± 5 for 50 s, respectively. The main wash (repeated three times) was carried out by DMF and alternatively by Dichloromethane (DCM), each in between deprotecting and coupling steps. Starting the synthesis, 0.2 M Fmoc-Lys (Biotin)-OH solution (prepared via supplier Protocol) was coupled with H-Rink amide resin via standard coupling reaction. The reaction, assisted by 0.5 M DIC and 1 M Oxyma, was carried out in an SPPS synthesizer at 90 °C ± 5 for 110 s, followed by 75 °C ± 5 for 15 s, with alternating steps of washing with DMF and DCM. After confirming the coupling by Kaiser Test, the de-protection was performed to remove Fmoc group. The second coupling reaction was performed using the same concentration (0.2 M) of 1-pyrene butyric acid in DMF, whereas the condition of reactions remained the same. Finally, the Kaiser test was performed again to ensure the success of the coupling reaction. The final product was dried in a desiccator for 1.0 h. The product was cleaved from the solid support by a cleavage solution containing trifluoroacetic acid (TFA): triisopropylsilane (TIS): H_2_O (95:2.5:2.5). The cleavage was performed by stirring the cleavage solution for 2.0 h at room temperature. Finally, the cleavage solute was filtered and separated from resin under the flow of pure nitrogen. Afterward, the product was precipitated in cold diethyl ether and lyophilized using Labconco Lyophilizer. The mechanism of reactions is explained in detail in Supplementary Section 1 (Figure S2). As explained, the confirmation of coupling and deprotection was carried out at each stage by the Kaiser Test, with results shown in Supplementary Figure S3. The final product was characterized (via UV spectroscopy) to confirm the conjugation of Pyrene into our supporter assembly. A clear peak at 335 nm was observed (Figure S4) in our supporter molecule, confirming the conjugation of Pyrene moiety. Finally, an optimized concentration (1 nM) of this supporter molecule was used to avoid over stacking and loss of material during functionalization. The concentration of the supporter construct was calculated using Beer’s Lambert law by measuring the absorbance at 335 nm.

### 2.3. SA Solution Concentration

The solid un-conjugated SA was purchased from Thermo Fisher scientific and a solution of 100 µM was prepared, followed by serial dilutions using distilled water. The concentration was confirmed by measuring the absorbance of the solution (at 280 nm) using a Bio spectrophotometer (Figure S5) and calculating the concentration via Beer’s Lambert law.

## 3. Results and Discussion

This section is divided by subheadings to provide a concise and precise description of the experimental results, their interpretation, as well as the experimental conclusions that can be drawn.

### 3.1. Gate-Modulated Electric Transport of WSe_2_ FET

The back gate voltage was applied to the bottom of the Si substrate, and the fixed V_DS_ was applied at the source electrode while keeping the drain electrode grounded. The pristine WSe_2_ device showed thresholds at gate voltages of *V_g_* > 10 V and *V_g_* < −20 V, which is attributed to electrons and holes, respectively, as shown in Figure 2c. The current level at positive gate voltage is higher, so the density of electrons is greater than holes, and the *n*-type nature is observed prominently. As the source-drain voltage is increased from 0.5 V to 1.5 V, the output current also increases. The electric transport of the charge carriers through the WSe_2_ device is regulated with the applied gate voltage. The charge carrier density of the electrons and holes is estimated by Equation (1).

(1) $$n={\;q}^{-1}C_{g}\left|V_{th}–V_{g}\right|$$

Here, q is the electron (hole) charge of one electron (hole), *V_g_* is the gate voltage, *V_th_* is the threshold voltage and *C_g_* is gate capacitance. The monolayer’s thick WSe_2_ sheet exhibited an excellent charge carrier density of 3.2 × 10^12^ cm^−2^ for electrons and 8.85 × 10^11^ cm^−2^ for holes. The output characteristics (I-V curves) of the pristine device are shown in Figure 2d at various gate voltages.

A higher current appears at positive gate voltages, while the current is suppressed at negative gate voltage. This is attributed to the *n*-type nature of the WSe_2_ sheet, in which the majority of charge carriers (electrons) flow at the positive gate voltage, while only minority charges can flow at the negative gate voltage [43,44].

### 3.2. Raman Spectra Analysis of WSe_2_ FET

**Figure 3:**
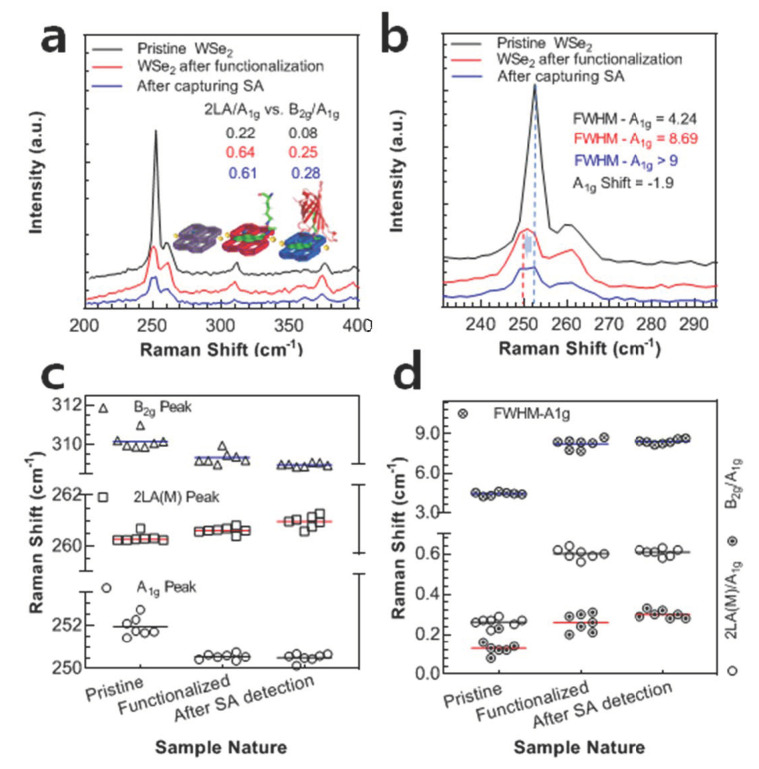
Raman spectra analysis of WSe_2_ FET device. The relative shifting and ratios of Raman peak after each device operation. (**a**) The spectra represent the intensity ratio of pristine, after functionalization and after capturing SA at 1 pM concentration. It can be seen that the intensity ratio of defects peak increases after the functionalization step. However, a negligible ratio change can be seen upon capturing SA, owing to its minute concentration used in the measurement. (**b**) The enlarged spectra of main resonance peak represent its shifting after functionalizing. The negative shift depicts the clear *n*-doping of WSe_2_ upon functionalization. Additionally, the FWHM value is increasing after functionalizing, indicating the loss of crystallinity in WSe_2_. (**c**) The relative position of the Raman peak and their shifting after functionalization is shown. The Line is representing the MEAN of seven (7) measurements. (**d**) The peak amplitude ratios and FWHM of WSe_2_ FET upon SA detection followed by its functionalization. The Line is representing the MEAN of seven measurements.

As explained earlier, Raman spectroscopy was performed using a 532 nm excitation laser and 100× magnification objective at each stage of device testing. Firstly, to ensure the high-quality monolayer of the WSe_2_ nanosheet, the spectra were measured in a span of 200–400 nm. The pristine/bare WSe_2_ exhibits a good intensity A_1g_ peak at ~252 cm^−1^, owing to out-of-plane vibrations, as shown in Figure 3a. However, a small enhancement peak at 260 cm^−1^ can be seen, which can be designated as 2LA(M). This enhancement peak can be attributed to the defects and disorders in the lattice while growing WSe_2_ [45,46]. The peak positions were confirmed by measuring the Raman spectra at seven different places and plotting their mean, which can be seen in Figure 3c. However, the relatively small peak ratio (0.22) of desired A_1g_ peak and 2LA(M) peak represents the high quality of defect-free WSe_2_ material. Moreover, the full-width half minimum (FWHM) of the A_1g_ peak comes to be 4.24, confirming a high level of crystallinity compared to possible values in the literature [46]. The high value of FWHM indicates the loss of crystallinity and high level of defects [47,48,49].The peak ratios and FWHM of A_1g_ peak were also calculated using Gaussian fit and plotted in Figure 3d, representing the consistency in measurements. Furthermore, a small peak at ~310 cm^−1^ position can be seen, which can be designated as B_2g_ resonance mode of WSe_2_ [50]. This mode of resonance is only active in the bi-layer region reflecting the presence of inter-layers interaction [51,52]. However, when viewing the WSe_2_ as a perfect monolayer, the ratio of this peak to the intended one (B_2g_/A_1g_) is 0.08, which is negligible. The peak ratios are calculated using other repeated measurements to avoid any possible error.

After functionalization of the device with our Pyrene-based supporter molecule, the Raman spectra were recorded, and it can be seen that A_1g_ peak at ~253 cm^−1^ is shifted to −1.9 cm^−1^, Figure 3b. This shift can be attributed to the high negative charge of the Pyrene molecule, since it has a lot of negative charges causing a push of electrons away from the plane [18,19,53,54,55]. This shifting in peak position can be consistently seen in Figure 3c, where the position was decreased, even after repeated measurements. Moreover, it can be seen that the ratio of resonance peak and enhancement mode (2LA/A_1g_) is increased from 0.22 to 0.64, indicating the functionalization of the device channel with our Pyrene-based supporter molecule. However, this increase cannot fully justify the increased number of layers upon functionalization [50]. Hence, the peak ratio of the second enhancement-mode was calculated, i.e., B_2g_/A_1g_. This ratio also represents a significant increase (0.20) compared to that of the pristine WSe_2_ peak ratio (0.08), Figure 3b. Moreover, the FWHM of A_1g_ peak also significantly increased from 4.24 to 8.69, representing a clear loss in crystallinity due to the attachment of the supporter molecule. This loss in crystallinity can be observed even after repeated measurements of FWHM, as shown in Figure 3c. Moreover, the AFM can be seen after functionalizing the device, Supporting Figure S6.

### 3.3. Sensitivity Test

**Figure 4:**
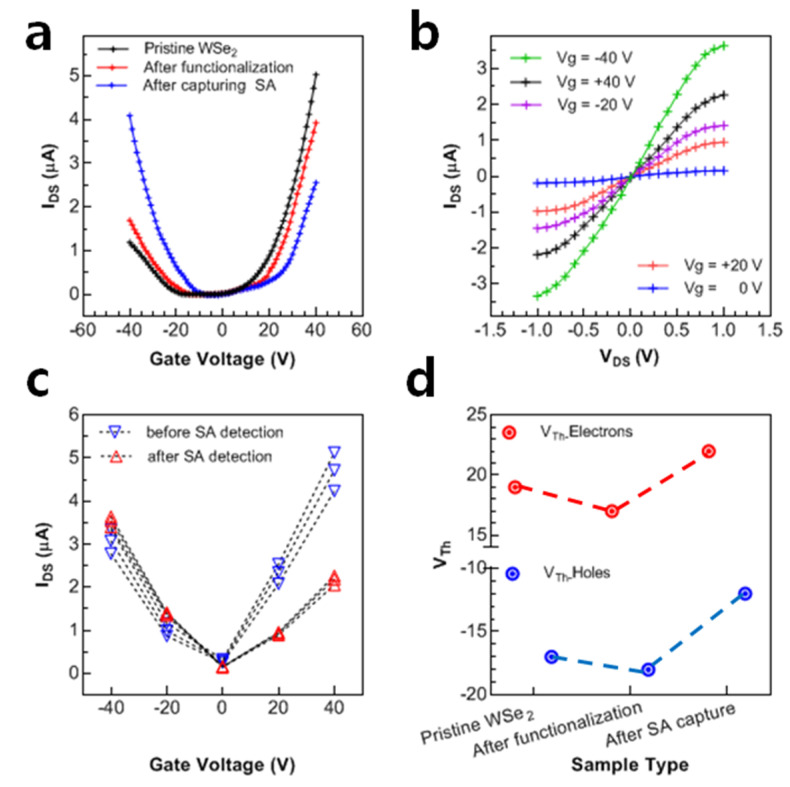
Electrical property analysis of WSe_2_ after capturing SA at 1 pM. (**a**) The transfer characteristics of the device at various stages of device operation. A minor shifting can be observed in the threshold voltage of pristine WSe_2_ upon functionalization. (**b**) The output characteristics of the device after capturing SA. Owing to the high sensitivity of the device, a large shift can be seen even after detecting the SA at a small concentration (1 pM). (**c**) The figure depicts the resolution of currents before and after detecting SA w.r.t. various gate voltages. (**d**) The shift in threshold gate voltage can be observed. It is clear that the current due to holes increases only after capturing SA.

The monolayer WSe_2_ FET device is utilized for the detection of the selected protein (SA) in the solution phase. The in-plane hexagonal nature of the WSe_2_ sheets provides an excellent plate form for the Pyrene ring, which is easily attached over its surface via π-π bonding, as shown in Figure 1c. The V_DS_ is adjusted to a fixed value of 0.5 V before device functionalization. For the functionalization of the WSe_2_ FET device, a 2.5 µL of supporter solution was drop cast on the channel by using a micropipette. Figure 4a exhibits the transfer curves of the device after its functionalization (red) and its comparison with the pristine state (black). As the Pyrene rings from the supporter molecules attached to the device, it showed a minor shift in its threshold voltages, but a large shift in threshold voltage is observed after attaching the protein molecules, owing to the large size of the SA molecule (~5 nm). The blue line in Figure 4a represents the shifting of the transfer curve after the SA binding. This shift in threshold is possibly attributed to hole doping. The maximum electrons are occupied by the biomolecules (Pyrene + protein) and it upsurges the current due to the holes [16,56,57]. After detection of the protein molecules, the I–V curves at various gate voltages are illustrated in Figure 4b. These I–V curves also show that the majority of charge carriers are holes after the detection of the biomolecules. As the Pyrene and protein attach to the WSe_2_ sheet, it changes its nature from more *n*-type to more *p*-type. Figure 4c shows the current level of the gate voltage before and after the detection of SA. The shift in threshold voltage is also demonstrated in Figure 4d for the pristine, functionalized, and SA capturing WSe_2_ device. This threshold shift clearly illustrates that the current of holes is increased after SA capturing.

In addition to the above, the SA-captured device was exposed to Raman testing again, this time under the same conditions as when it was functionalized, to observe the device’s behavior and compare it to earlier phases. Here, we can again observe a slight negative shift in the main resonance peak (A_1g_) due to the capturing of SA at a very low concentration, i.e., 1 pM. This shifting of first resonance and enhanced resonance (2LA) peaks are slight; however, the shift in the second resonance peak (B_2g_) can be observed in Figure 3c, under repeated measurements. Moreover, the second resonance to first resonance peak ratio (B_2g_/A_1g_), which represents the multi-layer interaction, is increased from 0.20 (before capturing SA) to 0.28. Furthermore, the FWHM of the main resonance peak broadens slightly, owing to a small amount of SA.

### 3.4. The Real-Time Response of WSe_2_ FET

**Figure 5:**
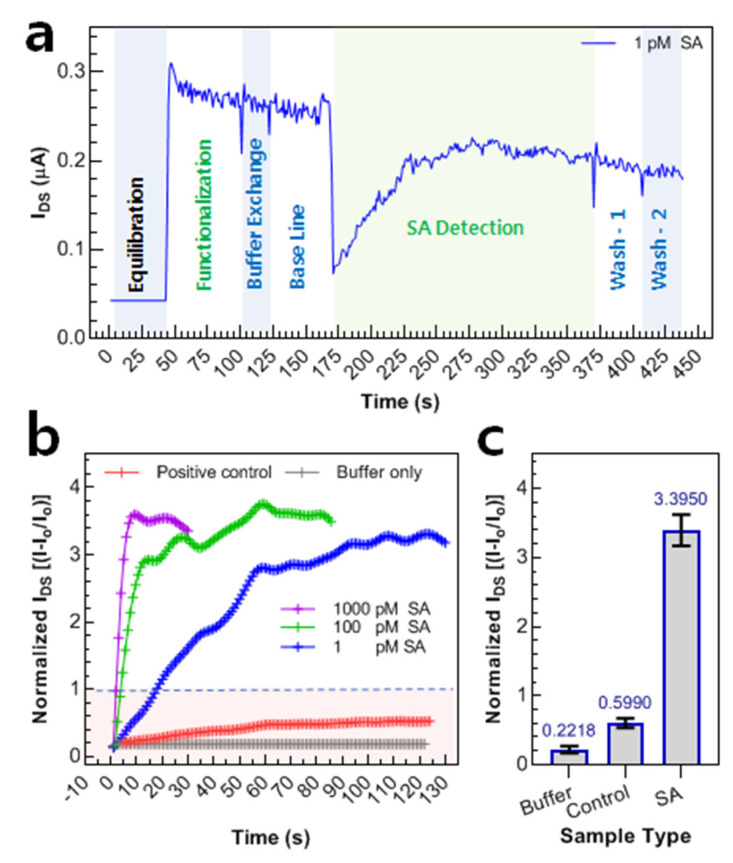
Real-time response of WSe_2_ FET towards SA at various concentrations. (**a**) The level of current at each stage of device operation can be seen. The measurement of SA was followed by equilibration, functionalization, and washing with solution buffer to create a baseline for measurement. A sharp increase in current can be seen upon functionalization owing to the high charge transfer from Pyrene moiety of supporter molecule to WSe_2_ sheet. It can be seen that the whole device operation lasts for ~5 min, making the device fit to be used in POC-based diagnosis. (**b**) The real-time response of the device at various concentrations of SA and towards control. The I_DS_ measured during each step was processed to generate the data. Owing to the sensitivity and robustness of our device, 95% of the ultimate response can be seen within 2 min for 1 pM SA concentration. The red shades represent the 5*STDEV values which mean concentration down from 1 pM can also be detected by sacrificing time. (**c**) A comparison of Normalized I_DS_ for the target (SA) and control (BSA) sample can be seen. The system shows a clear gap in the current ratio only after interacting with our target protein (SA).

Finally, to evaluate the robustness of prepared WSe_2_ FET, real-time testing was performed using a KeysightB1500-A semiconductor parameter analyzer. The input voltage was fixed to a small value of 0.2 V, and the output current was recorded as a function of time. As a first step, the equilibration was carried out to create an initial baseline and ~0.042 µA of the current was recorded. For the device functionalization, the supporter molecule containing solution (1 nM) was drop cast. As the Pyrene rings of the supporter molecule are attached to the WSe_2_ sheet, a large current is observed because of the charge transfer from the Pyrene ring to the WSe_2_ sheet. As functionalization occurred, the current was saturated (0.253 µA) within a min and there was no more increase in its value. This was marked as the functionalization of the WSe_2_ FET devices. Our receptor functionalized the channel surface in a highly self-oriented manner due to the large span of ring components attached to its N-terminal. After the current was leveled-off, the device was washed with solution buffer to wash away the unbounded supporter molecules. After a little fluctuation, it can be seen that the level of current almost remained the same, i.e., 0.261 µA. To ensure a proper wash and create the baseline, a second wash was applied and noted the current, i.e., remained the same (0.257 µA). The level of current remained the same before and after washing, owing to the optimized concentration of supporter solution (1 nM). Finally, the solution containing 1 pM SA was drop cast, and the level of current was noted. It can be seen that the current reached its equilibrium position within 2 min. It is believed that the SA binds to biotin due to their strong non-covalent interaction between the 10 amino acidic resides, explained in [57]. Owing to the non-covalent interaction, the level of current was decreased and leveled-off to a level of 0.208 µA. Finally, to ensure that the signal readout was due to the actual binding of SA rather than just being on the surface, we double washed the device with the solution buffer and it can be seen that the level of current remained the same, as shown in Figure 5a. The SA concentration was varied from 1000 pM to its 10-dilution fold and the current was noted. To eliminate the device-to-device error, the normalized value of the current was calculated and plotted in Figure 5b. In order to test the selectivity of our device, a simple solution buffer was used as a negative control and BSA (66.4 kDa), being a similar molecular weight to our target protein SA (66 kDa), was used as a positive control. After its measurement, a minute current response can be observed, which is due to the unwanted stacking of BSA on the channel via physical forces. After considering the level of the response given by serum protein, we calculated the standard deviation and marked the confidence level of the device, the red zone in Figure 5b. The difference in the level of response plotted for an average of three measurements for each sample can be seen in Figure 5c, showing the consistency of the results.

## 4. Conclusions

In this research, we reported a precise and prompt protein detection system through the control of receptor protein orientation and effective distance from the channel, using a high-quality monolayer WSe_2_ substrate. The biotin for sensitive streptavidin targeting and Pyrene conjugated Lysine supporter construct for ordered orientation on Wse_2_ FET were combined via protein engineering. Under this platform, the target protein was detected within 2 min with 1 pM limit of detection without any laborious functionalization. Due to the high quality of our WSe_2_ and directional functionalization, the total time for device operation was reduced to ~5 min. The selectivity was tested by an experiment of serum protein of similar molecular weight that represented a negligible signal in our WSe_2_ FET sensor. Our sensitive substrate and novel construct to control the orientation of receptors in WSe_2_ can shed light on developing rapid and accurate sensor systems for the detection of various pandemic targets. Moreover, it may have potential applications for the electronics industry to fabricate efficient energy harvesting devices owning to a fast photo-response.

## Data Availability

Not applicable.

## Supplementary Materials

The following supporting information can be downloaded at: https://www.mdpi.com/article/10.3390/nano12081305/s1, Figure S1: Fabrication scheme; Figure S2: Reaction mechanism for the supporter molecule synthesis; Figure S3: Kaiser Test; Figure S4: UV-spectra of supporter construct, confirming the conjugation of Pyrene to Lys-Biotin by SPPS; Figure S5: UV-Visible spectra of SA solution; Figure S6: AFM after SA capturing by WSe_2_ FET.
