# Supplementary material for: Precise and Prompt Analyte Detection via Ordered Orientation of Receptor in WSe2-Based Field Effect Transistor
Source: Nanomaterials (Basel). 2022 Apr 11;12(8):1305. doi: 10.3390/nano12081305 (PMC9028725; doi:10.3390/nano12081305)
Supplement: Supplementary file 1 [file nanomaterials-12-01305-s001.zip › nanomaterials-1650883-supplementary.pdf]

nanomaterials

Supplementary Material

Precise and Prompt Analyte Detection via Ordered Orientation of Receptor in WSe_2-Based Field Effect Transistor

Muhammad Shahzad Zafar [1,4], Ghulam Dastgeer [2,*], Abul Kalam [3,5], Abdullah G. Al-Sehemi [3,5], Muhammad Imran [3,5], Yong Ho Kim [4] and Heeyeop Chae [1]

[1] School of Chemical Engineering, Sungkyunkwan University, Suwon 16419, Korea; mshahzad@skku.edu (M.S.Z.); hchae@skku.edu (H.C.)
[2] Department of Physics and Astronomy and Graphene Research Institute-Texas Photonics Center International Research Center (GRI–TPC IRC), Sejong University, Seoul 05006, Korea
[3] Department of Chemistry, Faculty of Science, King Khalid University, P.O. Box 9004, Abha 61413, Saudi Arabia; abul_k33@yahoo.com (A.K.); agmsq@kku.edu.sa (A.G.A.-S.); imranchemist@gmail.com (M.I.)
[4] SKKU Advanced Institute of Nanotechnology (SAINT), Sungkyunkwan University, Suwon, 16419, Korea; yhkim94@skku.edu
[5] Research Center for Advanced Materials Science (RCAMS), King Khalid University, P.O. Box 9004, Abha 61514, Saudi Arabia
* Correspondence: gdastgeer@sejong.ac.kr; Tel.: +82-10-8343-1889

Device Fabrication and Capturing the Protein

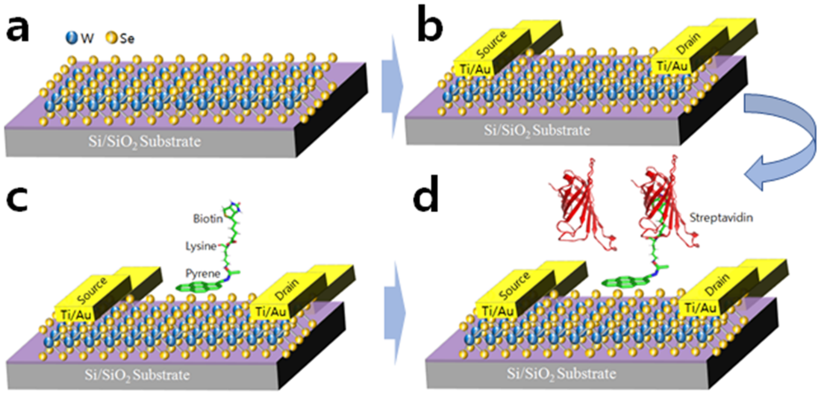

Figure S1: The flow chart represents a step-by-step fabrication and operation of the WSe_2 FET. **(a)** A monolayer WSe_2 sheet is transferred over the Si substrate after mechanical exfoliation. **(b)** Using thermal deposition, metal electrodes (Ti/Au) were deposited over the monolayer WSe_2 sheet. **(c)** Functionalizing the device by a Pyrene-based supporter molecule. **(d)** The detection of SA in the solution phase.

Mechanism of Reaction; For Supporter Molecule Synthesis

The mechanism of reactions while making a Pyrene-based supporter molecule can be explained on the basis of Figure S1, shown below. The H-Rink amide resin comes de-protected, so the NH_2 group is available for coupling reaction, mentioned in red. When the Fmoc protected Lysine_Biotin molecule was coupled via standard coupling conditions, it makes a covalent bond with –COOH group, mentioned in green. However, at this stage, the –NH group, mentioned in YELLOW is Fmoc protected. After washing the resin, the de-protection reaction was conducted under the standard conditions, men-

tioned above. The Fmoc group is removed and –NH becomes –NH_2, and becomes available for a new coupling reaction as N-terminal. Finally, when 1-pyrene butyric acid was added via standard coupling reaction, it attached on –NH2 and confirmed by Kaiser Test again, shown as step no. 3. Finally, the cleavage was done by standard cleavage solution, and polymeric resin is eliminated, step no. 4.

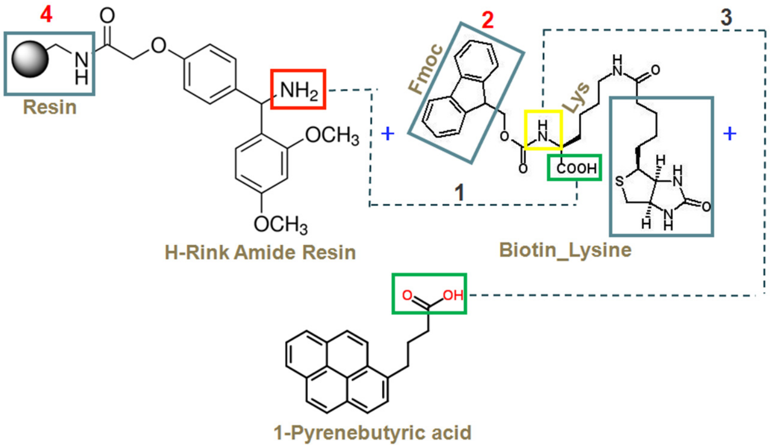

Figure S2. Reaction mechanism for the supporter molecule synthesis; 1 & 3 = coupling reaction, 2 = de-protection, 4 = cleavage.

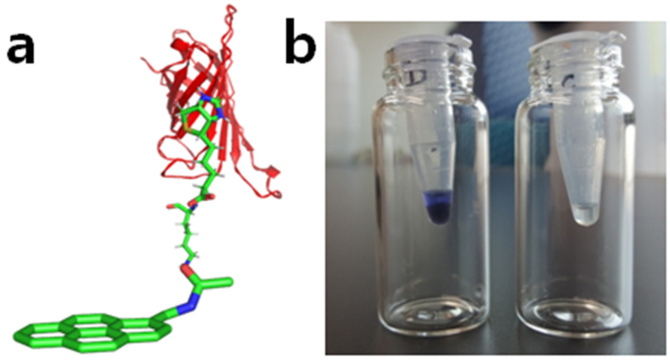

Figure S3. Kaiser Test. The violet color of resin beads is showing that de-protection is done. The colorless beads represent the successful coupling reaction.

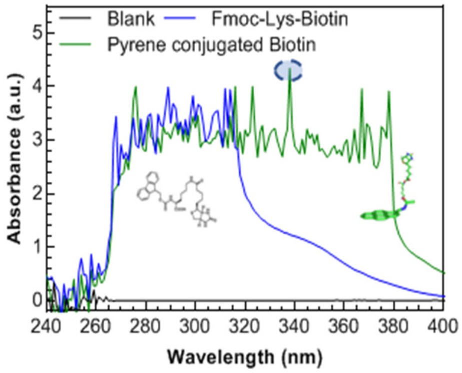

Figure S4. UV-spectra of supporter construct, confirming the conjugation of Pyrene to Lys-Biotin by SPPS. A sharp peak at ~335 nm shows the presence of Pyrene.

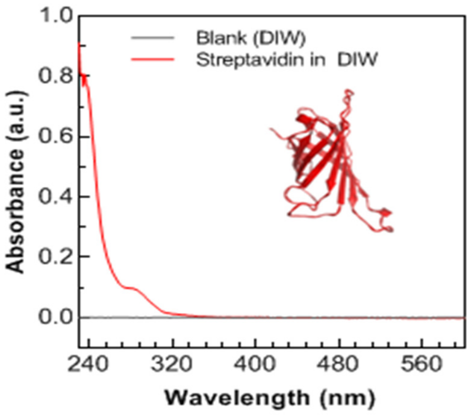

Figure S5. UV-Visible spectra of SA solution, confirming the concentration while making dilutions. The concentration was calculated by measuring the absorbance at 280 nm and using Beer's Lambert Law.

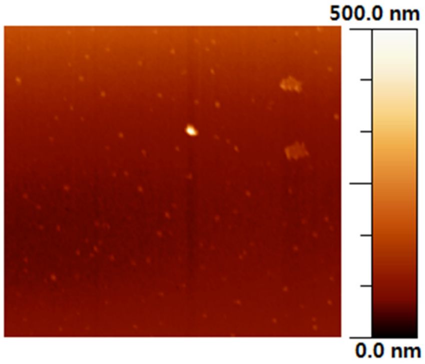

Figure S6. AFM after SA capturing at 1 pM. The SA molecules are observed clearly in the form of dots during the AFM analysis of the WSe_2 sheet. These dots are the indication of captured SA molecules by WSe_2 FET.
